# Primary Ewing's Sarcoma of Frontoparietal Bone with Major Soft Tissue Extension: An Unusual Presentation and Review of the Literature

**DOI:** 10.1155/2012/713836

**Published:** 2012-10-31

**Authors:** Anshu Gupta, Sachin Bansal, Sujata Chaturvedi

**Affiliations:** Department of Pathology, Institute of Human Behavior and Allied Sciences, Dilshad Garden, Delhi 95, India

## Abstract

An 11-year-old girl presented with progressively increasing swelling in scalp of 8-month duration with no neurological deficit. Local examination showed a hard swelling that seemed to be arising from frontal bone. General and systemic examination was normal. MRI revealed a well-defined lytic lesion in left frontoparietal bone with a subgaleal component. The patient was operated upon and excision of tumor with reconstruction of skull was done. Histopathological examination showed a monomorphic small round cell tumor of bone infiltrating into the subcutaneous tissue. Immunohistochemical stain showed diffuse immunopositivity for MIC-2 in tumor cells, thus final diagnosis of Ewing's sarcoma was made. The patient was kept for follow up for 3 months and had no symptoms.

## 1. Introduction

Ewing's sarcoma is most commonly seen in children and young adults with a peak incidence in the second decade of life. It most commonly arises in long bones of the extremities (predominantly femur) and pelvis [1]. Primary Ewing's sarcoma of the cranial bone is rare and contributes about 1% of all Ewing's sarcoma [2]. Temporal bone is most commonly affected followed by frontal and parietal bone [3]. Considering its unusual site and soft tissue extension, we report a case of Primary Ewing's sarcoma of frontoparietal bone with soft tissue extension.

## 2. Case Report

**Figure 1:**
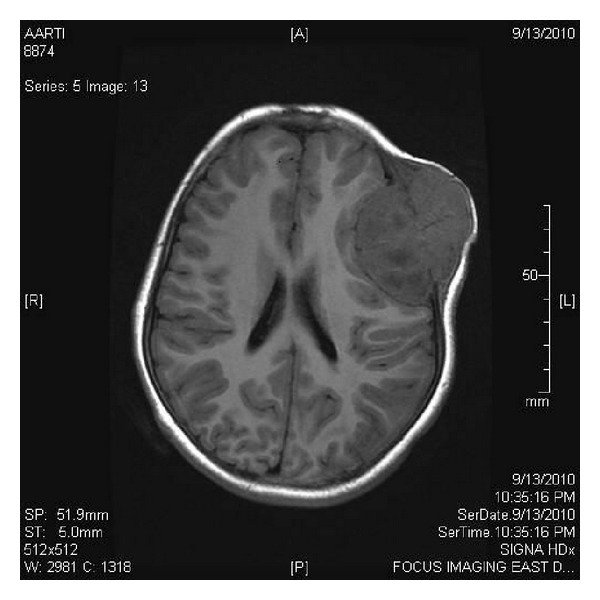
Postcontrast T1 weighted MRI showed intense, homogenously enhancing intracranial mass causing destruction of bone, with extradural soft tissue extension.

An 11-year-old girl presented with painless swelling in left frontoparietal region of scalp from 8 months and progressively increasing in size. The mass was detected incidentally when she had a fall. There was no history of headache, vomiting, fever, and seizure or any focal neurological deficit. Local examination revealed a hard mass of 50 × 48 mm in size with normal overlying skin. She has no sensory and motor deficit. Her general and systemic examination was normal. Blood investigations were normal. CT scan shows a well-defined lytic lesion in left frontoparietal bone. On MRI, a lytic lesion in left frontoparietal calvarium was seen with sun ray periosteal reaction, measuring 60 × 53 mm in size with a subgaleal and epidural component causing bulking of parenchyma (Figure 1). The patient was operated and excision of left frontal tumor with reconstruction of skull was done. Intraoperative frozen section revealed monomorphic small round cells arranged in clusters and scattered singly. Diagnosis of a malignant round cell tumor was made.

**Figure 2:**
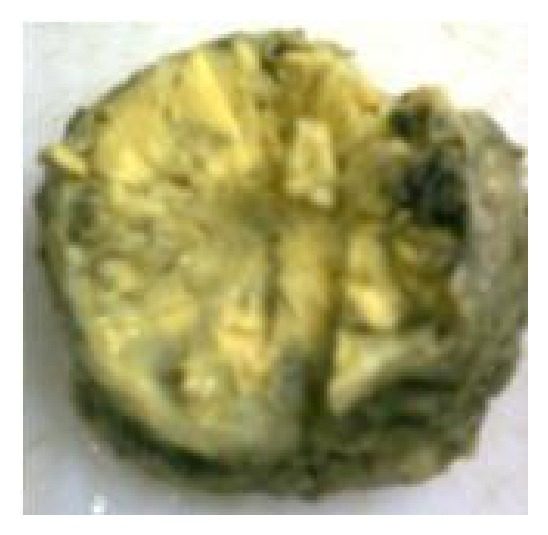
Gross specimen revealing well-circumscribed tumor with gray white to yellow gelatinous cut surface.

**Figure 3:**
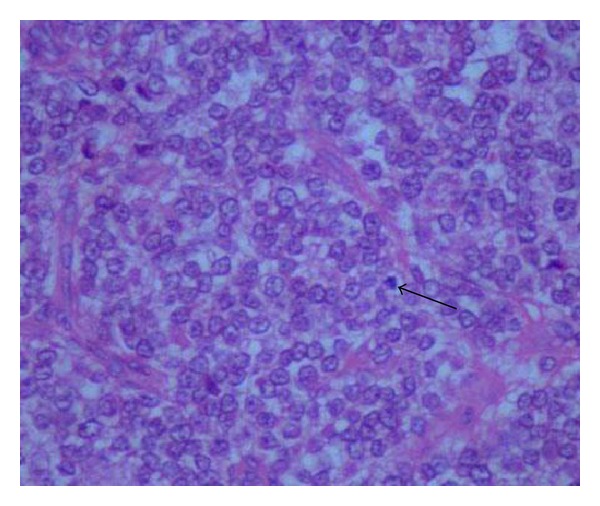
Microsection showing lobular arrangement of malignant round tumor cells with abundant vacuolated cytoplasm. Atypical mitosis also seen. Haematoxylin and Eosin (H&E) stain × 40x.

The tumor was sent for histopathological examination. Grossly, the specimen consists of one large grayish brown soft tissue attached to a flat bony fragment measuring 7.0 × 7.0 × 4.5 cms. External surface of the soft tissue was smooth and partially encapsulated. Cut surface of the soft tissue was gray white to yellowish gelatinous and showed few hemorrhagic areas also (Figure 2). On microscopic examination, section revealed a monomorphic round cell tumor arranged in lobular, trabecular, and micro- and macrofollicular pattern with eosinophilic secretion in the lumen (Figure 3). Tumor cells had round nuclei with stippled chromatin, prominent nucleoli, and thick nuclear membrane. Cytoplasm was moderate in amount and vacuolated. Connective tissue septae with fine blood vessels were seen throughout the tumor. Mitosis was 0–2/hpf. There was also infiltration of tumor cells in the surrounding fibroadipose tissue. Section examined from bone showed bony trabeculae and bone marrow revealing marked fibrosis and infiltration by tumor cells. On Periodic Acid Schiff (PAS) stain, tumor cells were negative. On immunohistochemistry, tumor cells were immunopositive for MIC-2. Keeping in view the morphological and immunohistochemical profile, a final diagnosis of Ewing's sarcoma of bone was made.

## 3. Discussion

Ewing's sarcoma involving the skull is rare and occurs in less than 1% of cases [2]. Commonest site of Primary Ewing's sarcoma is temporal bone followed by parietal and occipital bone. Sphenoid and ethmoid bones are less commonly involved [3]. In published cases of Primary Ewing's sarcoma of the cranium, the most common symptoms reported at the time of diagnosis have been local swelling (as in our case) and associated headache. Patient with the skull base as the primary site may present with proptosis and various types of cranial nerve palsy. When there is, intracranial extension or involvement of neural structures there may be features of raised intracranial pressure as well as focal neurological deficit [4]. In our case there is no focal neurological deficit.

On plain X-ray many patients may show areas of bone destruction as in our case, with irregular poorly defined margin. The most common CT finding is isodense lesion with marked heterogenous enhancement. Biopsy is essential for definitive diagnosis. In our case, the histological diagnosis is made by examining the morphology and immunohistochemical profile of tumor cells [5].

The main differential diagnosis of tumor involving the skull with adjacent soft tissue extension in children would include metastatic neuroblastoma, PNET, chordoma, and lymphoma. Less common differential diagnosis includes rhabdomyosarcoma, osteosarcoma, meningioma, Langerhan's cell histiocytosis, desmoplastic small round cell tumor, plasmacytoma, and solitary metastasis. The differentiation between these may not be possible on light microscopy and require special stains and immunohistochemistry for final diagnosis. Although cytoplasmic glycogen content was considered to be important in differential diagnosis, in specimen fixed in formalin and embedded in paraffin, the glycogen may not be always demonstrated with PAS staining as in our case [6].

Primitive neuroectodermal tumor expresses neuronal marker such as synaptophysin, neurofilament protein, nonspecific enolase or S-100. Lymphoma cells express CD_19_, CD_20_, and CD_2,5,8_. Chordoma express strong positivity for Pan CK and Epithelial membrane antigen [5]. MIC-2 is aspecific marker for Ewing's sarcoma and peripheral primitive neuroectodermal tumors [7].

Immunopositivity for MIC-2 confirmed the diagnosis in our case.

 Early diagnosis and treatment prior to metastasis is essential for long-term survival in patient with Ewing sarcoma. The disease is treated through multidisciplinary approach that includes surgery, chemotherapy, and radiotherapy. This patient was kept for follow up and had no symptoms. In conclusion, primary cranial Ewing's sarcoma is to be considered in the differential diagnosis in children with a tumor involving the skull with destruction of bone and the presence of extra axial soft tissue involvement. Primary Ewing's sarcoma is reported to have a better prognosis as compared to Ewing's sarcoma elsewhere.

## References

[1] Unni KK, Unni KK (1996). Ewing’s tumor. *Dahlin’s Bone Tumors: General Aspects and Data on 11087 Cases*.

[2] Steinbok P, lodmark O F, Norman MG, han KW C, Fryer CJ (1986). Primary Ewing’s sarcoma of the base of skull. *Neurosurgery*.

[3] Singh P, Jain M, Singh DP, Kalra N, Khandelwal N, Suri S (2002). MR findings of primary Ewing’s sarcoma of greater wing of sphenoid. *Australasian Radiology*.

[4] Desai KI, Nadkarni TD, Goel A, Muzumdar DP, Naresh KN, Nair CN (2000). Primary Ewing’s sarcoma of the cranium. *Neurosurgery*.

[5] Schmidt D, Harms D, Pilon VA (1987). Small-cell pediatric tumors: histology, immunohistochemistry, and electron microscopy. *Clinics in Laboratory Medicine*.

[6] Sim FH, Unni KK, Beabout JW, Dahlin DC (1979). Osteosarcoma with small cells simulating Ewing’s tumor. *Journal of Bone and Joint Surgery A*.

[7] Ambros IM, Ambros PF, Strehl S, Kovar H, Gadner H, Salzer-Kuntschik M (1991). MIC2 is a specific marker for Ewing’s sarcoma and peripheral primitive neuroectodermal tumors: evidence for a common histogenesis of Ewing’s sarcoma and peripheral primitive neuroectodermal tumors from MIC2 expression and specific chromosome aberration. *Cancer*.

